# Natural enamel caries, dentine reactions, dentinal fluid and biofilm

**DOI:** 10.1038/s41598-019-38684-7

**Published:** 2019-02-26

**Authors:** Laryssa de Barros Pinto, Maria Luiza Lima Alves Lira, Yuri Wanderley Cavalcanti, Eugênia Livia de Andrade Dantas, Maria Lúcia Oliveira Vieira, Gabriel Garcia de Carvalho, Frederico Barbosa de Sousa

**Affiliations:** 1 Master Program in Dentistry, Health Sciences Center, Federal University of Paraiba, Cidade Universitaria, 58051-900 João Pessoa, Paraiba Brazil; 2 Department of Clinical and Social Dentistry, Health Sciences Center, Federal University of Paraiba, Cidade Universitaria, 58051-900 João Pessoa, Paraiba Brazil; 3 Department of Morphology, Health Sciences Center, Federal University of Paraiba, Cidade Universitária, 58051-900 João Pessoa, Paraiba Brazil

## Abstract

It is believed that penetration of dentinal fluid into natural enamel caries (NEC) is negligible because of the barrier created by underlying sclerotic dentine, but there are conflicting evidences on whether dentine subjacent to NEC is sclerotic or demineralized. This study aimed at investigating the relationship between NEC, subjacent dentine reactions, modification of dentinal fluid, and composition of cariogenic biofilm formed on the NEC surface. Proximal NEC (PNEC) lesions of human permanent posterior teeth were included in five experiments. Histologically, microradiographic analysis with contrast solution (MRC) in dentine revealed a decreased proportion of sclerotic dentine and an increased proportion of deep dentine demineralization compared to the classical stereomicroscopic histological analysis based on dentin color and translucency. Real-time MRC and 3D optical profilometry, and 3D microtomographic analysis evidenced a facilitated transport of modified dentinal fluid towards PNEC lesions. Cariogenic biofilm formed *in vitro* on the PNEC surface presented lower amounts of insoluble and soluble matrix polysaccharides when 2% chlorexidine was inserted in the pulp chamber. In conclusion, this study evidenced that dentine subjacent to PNEC is mostly demineralized, providing facilitated pathway for dentinal fluid to penetrate into PNEC and alter the composition of the biofilm formed on the PNEC surface.

## Introduction

In sound teeth, enamel and dentine are permeable to water and ions, which are transported from the tooth surface to the pulp and vice versa, with normal enamel as the main barrier to such permeability^1–3^. In natural enamel caries (NEC), pores are enlarged, increasing permeability and making it possible even the transport (by capillarity) of fluid resins (infiltrants) from the enamel surface toward the body of the dried enamel caries lesion^4^. Transport of materials into NEC lesion from underlying dentine is believed to be negligible. This is based on evidences suggesting that, even before enamel demineralization reaches the enamel-dentine junction, dentine subjacent to NEC forms mineral crystals obliterating its main transport pathways (dentinal tubules), creating sclerotic dentine close to the enamel-dentine junction^5,6^. In line with this, current noninvasive treatment strategies (sealant, caries infiltration, and remineralization) for enamel caries rely only on the transport of materials from the enamel surface inward.

Most of the evidences on the sclerotic nature of dentine subjacent to NEC is based on the translucent aspect of dentine under stereomicroscopy^5,7,8^. Since the early applications of microradiography in teeth, a number of reports have provided evidences that translucent dentine can be either sclerotic or demineralized^9–14^, not to mention the microradiographic finding of sclerosis in dentine with a dark aspect under stereomicroscopy^10^. Recently, it was reported a high frequency of deep dentine demineralization (>50% of dentine) underlying NEC^14^. In cavitated natural dentine caries lesions, it was reported that tubules in sclerotic dentine are partially obliterated and that the surrounding intertubular dentine (another pathway for materials transport in dentine^3^; Shellis 2000) is demineralized^15^. Those evidences suggest the existence of facilitated transport pathways for dentinal fluid in dentine underlying NEC. This might imply in a facilitated transport (transport at a higher rate than that in sound dentin) of dentinal fluid into the pores of carious enamel, mixing up with enamel fluid and, then, with bacterial biofilm fluid, affecting NEC progression. Dental materials containing antibiotics (chlorexidine, for instance) and/or fluoride that come to contact such facilitated transport pathway underneath proximal NEC (PNEC) are possible candidate agents to affect PNEC progression. Such cases occur in teeth with PNEC in proximal surfaces and deep restorations in the occlusal surface. Thus, this study aimed at testing, in PNEC, the relationship between enamel and dentine reactions caries, dentinal fluid transport, and the composition of cariogenic biofilm formed *in vitro* on the PNEC surface.

## Materials and Methods

This was an experimental, controlled, *in vitro* study using teeth (premolars and third molars) donated by volunteers who signed an informed and free consent, as approved by the Ethical Committee on Research in Humans of the Hospital of the Federal University of Paraiba (Brazil) in accordance with the regulations of the Brazilian National Council on Health. More details on Materials and Methods can be found in the Supplemental Material.

### Proximal Natural Enamel Caries (PNEC) Lesions

Proximal natural enamel caries (PNEC) lesions, from premolars and third molars, had their biofilm removed by applying (for 30 s) a cotton pellet soaked with 1% sodium hypochlorite, washed with water, and then lesions with ICDAS score 0–3^16^ and Nyvad score^17^ of inactive were selected by a calibrated examiner in both ICDAS (Kappa of 0.89) and Nyvad (Kappa of 0.9) systems for enamel caries. All teeth were stored in aqueous solution of 0.02% sodium azide before use in the experiments.

### Transport of dentinal fluid towards the PNEC (Study I)

**Figure 1:**
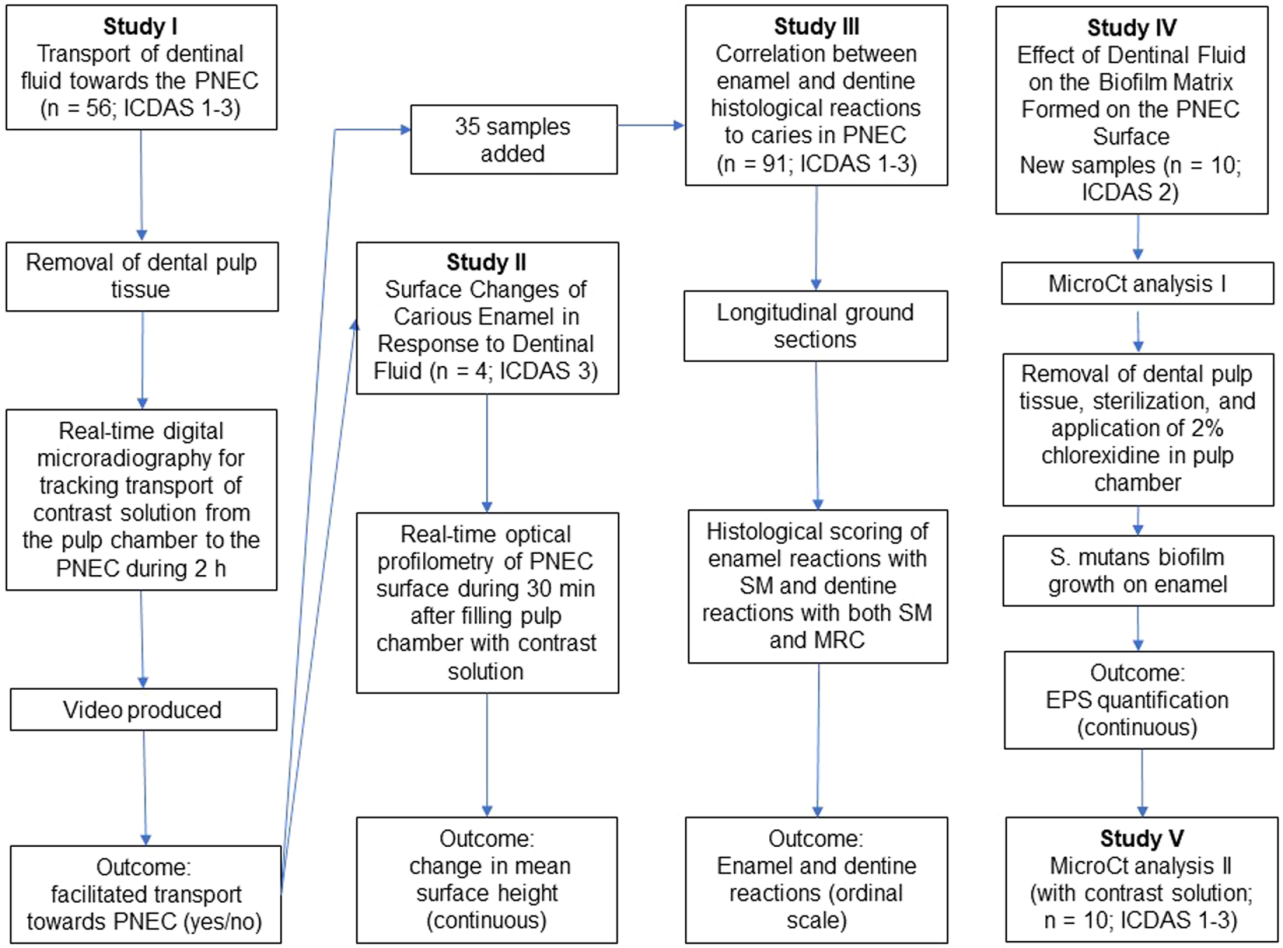
Flow chart of all experiments.

Here, it was tested the null hypothesis that a small proportion of teeth with PNEC present a facilitated transport of dentinal fluid from the pulp chamber to the PNEC Fig. 1. Teeth (n = 56; ICDAS scores 1–3) were transversally cut at the apical border of the coronal third of their roots, using a diamond disc mounted in a low speed dental motor under water irrigation. Then coronary pulp tissue was removed with the aid of a curette, Hedstroem type endodontic file, supplemented with irrigation with 1% NaOCl. With the aid of condensation silicon, each lesion was placed in a fixed position on the sample holder, perpendicular to the X-Ray source, and submitted to real-time digital microradiography (60 kV, 0.15 mA, Tungsten anode; PCBA Inspector, GE, Germany) before and after (at time intervals of 5 min, and then at 10 min intervals up to 2 h) the infiltration of a contrast solution (aqueous solution of potassium and mercuric iodide with refractive index of 1.47, pH 7.0; Thoulet’s solution)^14^ into the coronal pulp chamber. Time-series images resulted in a video, which was processed with pseudo-colors and then used for analysis of transport of dentinal fluid using a dichotomous outcome: with or without facilitated transport towards the PNEC. Comparisons were made among dentine regions in the same proximal side. At the end, the contrast solution was removed by immersing teeth in distilled water for 2 days.

### Surface Changes of Carious Enamel in Response to Dentinal Fluid (Study II)

Selected from the previous experiment, PNEC with ICDAS score 3 (n = 4) were investigated regarding enamel surface contour changes in response to the application of the contrast solution (Thoulet’s 1.47) in the pulpal wall of coronary dentine Fig. 1. Using a customized holder made with condensation silicon impression material, each tooth was placed in a fixed position on the stage of a 3D optical profilometer (CCI MP Profiler, Taylor Hobson, UK) so that an area on the PNEC surface could be imaged. Selected areas were tridimensionally imaged (with a 10X objective) before and after (at 5 min intervals up to 30 min) infiltration of Thoulet’s solution in the pulp chamber, at 50% of relative humidity and 22 °C. The outcome was the change in mean surface height (ΔMSH) across the center of field of view of the enamel surface during the 30 min interval. The method error was 1.18 μm, as determined by analyzing, at the same time intervals, 48 other tooth proximal surfaces (see Supplemental Material).

### Correlation between enamel and dentine histological reactions to caries (Study III)

In addition to teeth analyzed with real-time microradiography, another 35 proximal surfaces (total of 91 teeth) were analyzed regarding the correlation between enamel and dentine reactions to caries Fig. 1. We tested the null hypothesis that correlation between enamel and dentine reactions do not differ, regardless dentine reactions are analyzed under SM or microradiography with contrast solution. The main aspect investigated here was the difference between dentine histological aspects detected by classical SM and dentine histological aspects detected by microradiography with contrast solution. Longitudinal undemineralized ground sections (thickness of 1 mm) of tooth crowns were obtained by cutting (using diamond disc under water irrigation) and grinding (using lapping jig with silicon carbide paper) procedures intermittently with microradiographic analysis so that regions with deepest enamel and dentin demineralization could be preserved, as described before^14^, and a histological scoring system of enamel and dentine reactions (modified from Bjorndal & Thylstrup)^5^ was used. For enamel: demineralization in the outer ¼ of the enamel layer (E1), demineralization between ¼ and 2/3 of the enamel layer (E2), demineralization in more than 2/3 of the enamel layer (E3), demineralization reaching the enamel-dentine junction (E4), and demineralization reaching the enamel-dentine junction combined with enamel cavitation (E5). For dentine: no reaction (D1), sclerotic dentine (D2), demineralization restricted to mantle dentine (D3), demineralization up to the outer 50% of dentine (D4), and demineralization in the inner 50% of dentine (D5). A single examiner analyzed enamel reactions under stereomicroscopy (SM) only (Kappa of 0.8), and dentine reactions under SM (Kappa 0 f 0.89) and digital microradiography with contrast solution (MRC; according to Campos *et al*.)^14^ (Kappa of 0.9). After SM analysis, the same section was immersed in the contrast solution (2.0 ml in a 2.5 ml plastic sealed tube) for 24 h, and then microradiographed.

### Effect of Dentinal Fluid on the Biofilm Matrix Formed on the PNEC Surface (Study IV)

Here, it was tested the null hypothesis that the dentinal fluid did not affect the amount of extracellular polysaccharides in the cariogenic biofilm formed *in vitro* on the PNEC surface Fig. 1. A new set of teeth (ICDAS score 2; and Nyvad score of inactive; n = 10) was used to test the hypothesis that a modification of the dentinal fluid affects the composition of a cariogenic biofilm formed *in vitro* on the NEC surface. Teeth with fracture, crack lines, restoration, enamel defects, cavitated caries lesion, or internal resorption were excluded based on a baseline microCT analysis. After removal of pulp tissue as described before, teeth were cleaned using brushing and application of 1% NaOCl and water, and then stored in aqueous solution of 0.02% sodium azyde. Then, the root part was inserted and fixed in a plastic micro-tube, the tooth/plastic interface was sealed (see Supplemental Material), and a layer of acid resistant varnish was applied on the entire crown surface except on the area of the PNEC. The set of tooth in the sealed tube was sterilized in ethylene oxyde, and then the unprotected enamel surface was positioned upside down and submitted to *in vitro* cariogenic biofilm formation using *Streptococcus mutans* UA159, in medium with TYE and 1% sucrose (changed every 24 h) (see Supplemental Material), in four consecutive periods of 5 days. At the end of each period, biofilm was removed, the pulp chamber and crown were washed with distilled water for 5 min, and a new sterilization was performed. In this paired study, four dependent groups were included: PNEC and 2% chlorexidine in the pulp chamber filled (ECCh), PNEC and 0.9% NaCl in the pulp chamber (ECNaCl), normal enamel surface (on a surface opposite to proximal surface with PNEC) and 2% chlorexidine in the pulp chamber (NECh), and normal enamel and 0.9% NaCl in the pulp chamber (NENaCl). Each tooth provided data for each one of the four groups. Control area within any given tooth was the area that was neither affected nor subjacent to PNEC. After each 5 days period of biofilm formation, biofilm was removed and extracellular polysaccharides (EPS), both soluble and insoluble, were quantified following the procedure described recently^18^. More information can be found in the Supplemental Material.

### Analysis of subjacent dentine reactions using 3D microCT with contrast solution (Study V)

Teeth used in the biofilm formation experiment were later analyzed under microCT in order to track the path of dentinal fluid from the pulp chamber to the PNEC Fig. 1. Dentinal fluid was modified by infiltrating the Thoulet’s solution 1.47 in the pulp chamber for 24 h, then the excess of contrast solution was removed, and teeth were submitted to microCT analysis as described before. More information can be found in the Supplemental Material. A flow chart of all experiments is presented in Fig. 1.

### Statistical analysis

For penetration of dentinal fluid into dentine underlying PNEC, we tested the null hypothesis that the proportion of teeth with facilitated transport in dentine was small (small interpreted as a proportion of 20%, which was tested against the experimental proportion). A one-sample Z test, with 2-tailed 5% significance level, was used and Cohen H effect size and its confidence interval, and power were calculated^19^. The error of the surface profilometric analysis was calculated as described in the Supplemental Material. Correlations between enamel and dentine reactions were tested with Spearman correlation (95% CI, T test, and power) and the difference between correlation coefficients was tested using a T test, Cohen q effect size, and power^19^. The effect of dentinal fluid on the EPS of the cariogenic biofilm formed on the surface of PNEC was tested with repeated measures one-way ANOVA (for both soluble and insoluble EPS), followed by T test, and Hedge’s g, its 95% CI, and power were calculated. Test of normality of data, homogeneity of variances, and a priori sample size calculations are presented in Supplemental Material.

## Results

**Figure 2:**
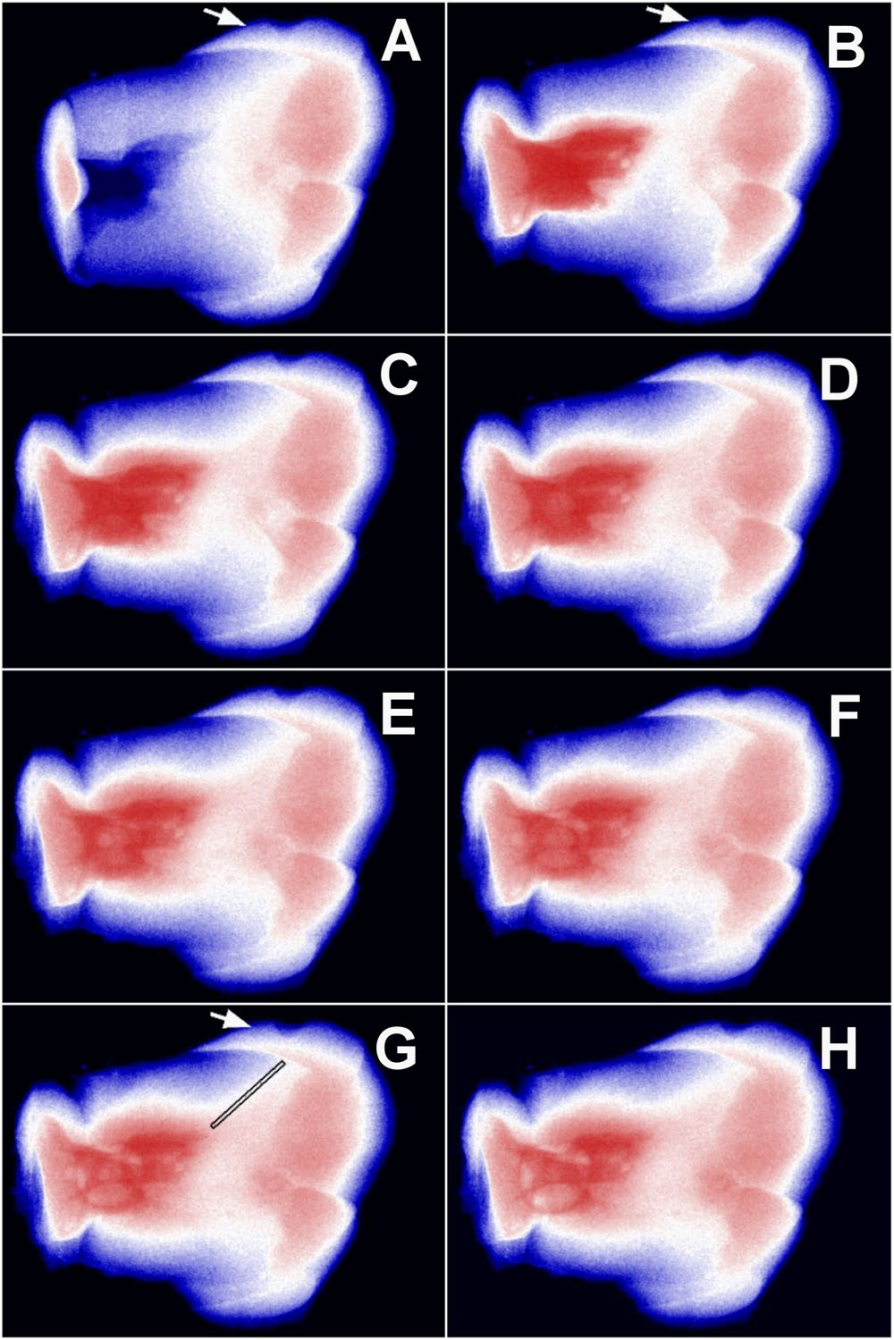
Time-series images sequence (up to 2 h) of the displacement of Thoulet’s solution from the pulp chamber towards the cavitated PNEC (ICDAS score 3; white arrow). A baseline image was obtained with no liquid in the pulp chamber. (**A**) After infiltration of Thoulet’s solution in the pulp chamber, new images were obtained after 5 min intervals. Sites of marked enamel and dentine changes are indicated by arrows (enamel; **A**,**B** and **G**) and rectangle (dentine; **G**).

**Figure 3:**
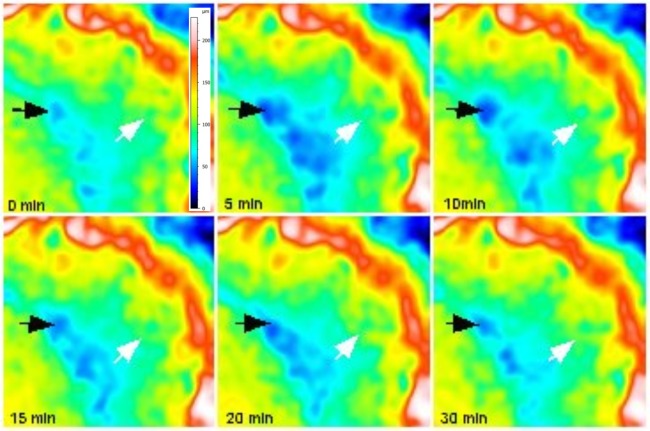
Time-series images sequence (0 min, 5 min, 10 min, 15 min, 20 min, and 30 min) of changes in surface topography of PNEC after infiltration of Thoulet’s solution in the pulp chamber. Colors indicate variations in surface height (see color scale in A, which is valid for all images). Areas with the most pronounced surfaces changes are indicated by arrows.

Using real-time 2D microradiographic analysis, a facilitated transport of dentinal fluid from the pulp chamber towards the PNEC was detected in 42.86% of the lesions, being statistically different from the expected proportion of 20% (p = 0.0081) with a medium effect size (Cohen H of 0.51), and power of 84.3%. In lesions with ICDAS score 3, the contrast solution (dentinal fluid) penetrated into the body of the PNEC (Fig. 2). In such lesions, profilometric analysis of surface changes in response to displacement of dentinal fluid revealed that all of the 4 lesions presented variations in surface height (in μm) after infiltration of the contrast solution into the pulp chamber (Fig. 3 and Supplemental Material Fig. 5).

**Figure 4:**
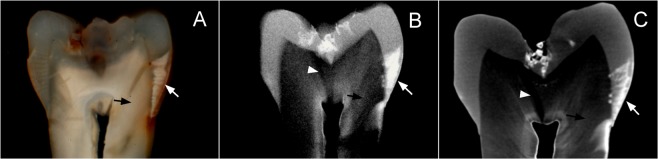
Typical histological aspects of enamel and dentine reactions to caries under SM (**A**) MRC (**B**) and microCT slice with contrast solution. (**C**) Enamel demineralization (white arrow) reaching the enamel-dentine junction is detected in all three techniques. For dentine reactions, while dentine (black arrow) seems normal under SM (SM score 1), deep dentine demineralization is detected in both MRC and microCT with contrast solution. White arrowhead shows sclerotic dentine under MRC and microCT.

Correlation between enamel and dentine reactions to caries was high when using SM for analyzing dentine reactions (Spearman r of 0.654, 95% CI of 0.74–0.54, p < 0.0001), but decreased when MRC was used for analyzing dentine reactions (0.238, 95% CI of 0.39–0.07, p = 0.02) (Supplemental Material Fig. 6). The difference between correlation coefficients was statistically significant (p < 0.00001; with a large effect size Cohen q of 0.54, 95% CI of 0.61–0.22, and power of 97.4%). The distributions of histological scores in dentine differed from SM to MRC (Supplemental Material Table 1), and the difference was mostly due to a much higher proportion of lesions with deep dentine demineralization detected when MRC was used (p < 0.00001; large effect size Cohen H of 1.75, and power >99.9%). Typical histological features of PNEC under SM and MRC are shown in Fig. 4, where SM shows dentine demineralization in <50% of dentine and MRC shows deep dentine demineralization reaching the pulp-dentine border.

The null hypothesis that the dentinal fluid subjacent to PNEC did not affect the composition of the cariogenic biofilm formed on the PNEC surface was rejected (ANOVA: (i) soluble EPS: p = 0.0002, effect size η^2^ = 36.8%, and power = 97.8%; (ii) insoluble EPS: p = 0.005, effect size η^2^ = 30.0%, and power = 90.8%). The ECChl group resulted in the lowest amounts of both insoluble and soluble EPS, while 0.9% NaCl in the dentinal fluid underlying PNEC (tested just after the use of chlorexidine in the dentinal fluid) reduced EPS compared to groups where biofilm was formed on sound enamel, and, among the later groups, the difference was not statistically significant (Table 1).

**Table 1:** Results of the effect of dentinal fluid on the composition (amounts of soluble and insoluble EPS, in μg/mL) of *S.mutans* biofilm formed on PNEC surface.

	Groups			
	ECChl	ECNaCl	NEChl	NENaCl
Soluble EPS - Mean (SD)	152.76 (33.18)	289.98 (73.76)	703.33 (379.4)	936.30 (524.30)
Insoluble EPS - Mean (SD)	78.01 (60.66)	329.51 (53.15)	550.68 (231.91)	768.12 (583.96)
***Post hoc paired T tests Soluble EPS***				
**Groups compared**	**P value**	**Hedge’s g**	**95% CI**	**Power**
ECChl X ECNaCl	0.0002	1.92	3.14/ 0.69	0.998
ECChl X NEChl	0.0011	1.50	2.64/ 0.35	0.982
ECChl X NENaCl	0.0009	1.53	2.68/ 0.38	0.985
ECNaCl X NECChl	0.0115	1.18	2.27/ 0.08	0.910
ECNaCl X NENaCl	0.0044	1.26	2.37/ 0.15	0.940
NEChl X NENaCl	0.0474	0.73	1.77/−0.32	0.513
***Post hoc paired T tests Insoluble EPS***				
ECChl X ECNaCl	3.03 × 10^−6^	3.23	4.76/1.69	0.999
ECChl X NEChl	0.0002	1.95	3.17/0.72	0.998
ECChl X NENaCl	0.0059	1.13	2.22/0.04	0.890
ECNaCl X NECChl	0.0290	1.10	2.18/0.01	0.871
ECNaCl X NENaCl	0.0479	0.78	1.82/−0.27	0.572
NEChl X NENaCl	0.1976	0.44	1.46/−0.58	0.203

**Figure 5:**
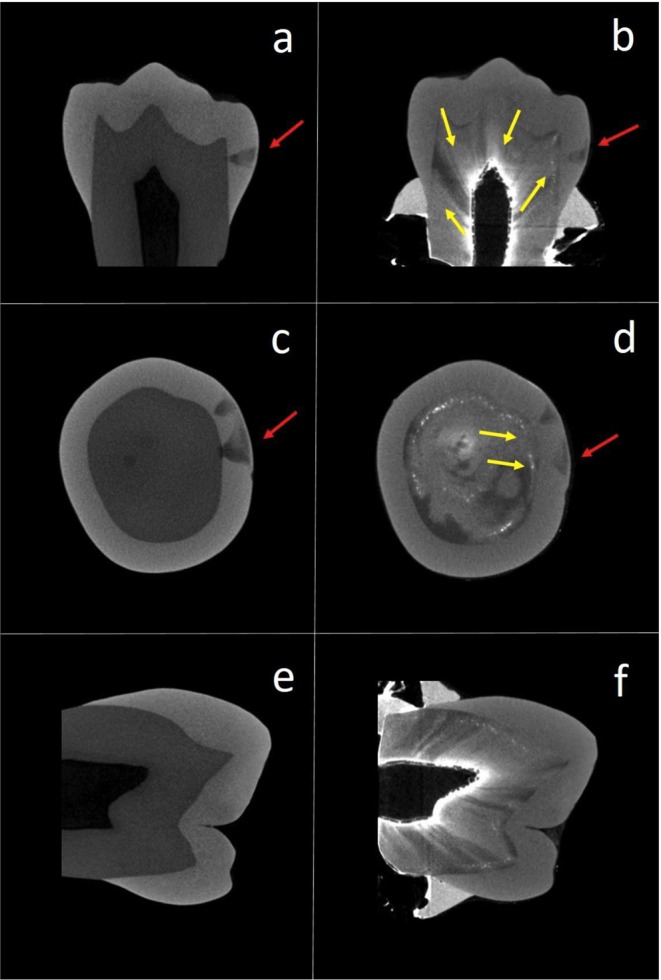
Typical histological aspects of enamel and dentine reactions to caries of a PNEC lesion analyzed under microCT. (**a**,**c** and **e**) show images of a microCT scanning obtained before the biofilm experiment. (**b**,**d** and **f**) show images of the same tooth scanned after the biofilm experiment terminated and after a 24 h-infiltration of the contrast solution in the pulp chamber. Contrast solution evidenced a facilitated transport pathway from the pulp chamber across the dentine layer (arrows) up to the body of the PNEC. Reduction in the radiolucent PNEC area is shown after infiltration of the contrast solution.

In all teeth used in the *in vitro* biofilm formation, 3D microCT analysis detected that the radiographic contrast solution infiltrated in the pulp chamber penetrated all thee way through the dentine layer and eventually penetrated into the body of the PNEC (Fig. 5).

## Discussion

This study provides evidence that dentine subjacent to PNEC is mostly demineralized and offers a path for facilitated transport of dentinal fluid to the body of the PNEC, with important implications to pathogenesis and noninvasive treatment of PNEC. Dentine reactions analyzed here are parts of caries-affected dentine^15^. While implication to teeth with vital pulp and no restoration is very limited, due to the barrier effect of the odontoblast layer and their cell extensions into dentinal tubules, our results impact mainly teeth (either pulpless endodontically treated or with vital pulp and deep occlusal restoration) with PNEC in proximal surface and deep restoration in the occlusal surface.

Even with the overlapping of normal, sclerotic, and demineralized dentine in the 2D digital microradiograhic images of whole crowns, the real-time microradiographic tracking of the displacement of dentinal fluid provided evidence of a facilitated transport of the contrast solution in dentine that was consistent with subsequent histological analysis. The penetration of contrast solution into the body of PNEC was detected only in cavitated lesions (ICDAS score 3). This can be explained by two factors: the competition with air discharge from the body of the PNEC lesion, and the presence of a histological surface layer in lesions with ICDAS scores 1–2. In porous materials such as NEC, penetration of liquid into the pores in the bottom part displaces air towards the outer surface^20^. The exit of air from PNEC might be hindered by the presence of a surface layer (expected to be preserved in lesions with ICDAS score 1–2, but discontinued in ICDAS score 3) because permeability in this layer is lower than that in the body of the lesion^21^.

Results from real-time tracking of dentinal fluid in whole crown are consistent with the high proportion of deep dentine demineralization subjacent to PNEC detected with MRC in ground histological sections. While the correlation between enamel and dentine reactions to caries was high when SM was used to detect dentine reactions, consistent with previous reports using SM^5^, the correlation decreased (with a high effect size) when MRC was used to detect dentine reactions to caries. The difference was mainly due to a higher proportion of deep dentine demineralization (>50% of dentine) detected with MRC. This difference confirms similar results in occlusal NEC^14^. The lower proportion of PNEC with deep dentine demineralization could be explained by the ambiguous nature of the aspect of dentine under SM, which underestimates the severity of NEC^12,14^. While translucent dentine under SM is interpreted as sclerotic dentine^5,7,8^, various studies reported microradiographic evidence that translucent dentine under SM can be either sclerotic or demineralized^9–14^, and sclerotic dentine (detected by microradiography) can also be either translucent or dark under SM^9^. The contrast solution used here penetrates more in demineralized dentine than in sound and sclerotic dentine^14^, thus enhancing the identification of the demineralized part of caries-affected dentine. The misleading interpretation of translucent dentine as sclerotic dentine contributed to the lack of other previous studies on transport of materials across dentine subjacent to PNEC.

During *in vitro* infiltration of Thoulet’s solution 1.47 (the same as our contrast solution) into ground sections of NEC, it has been shown that an intense displacement of air by the infiltrating solution is still occurring up to 2 h after immersion of NEC in the solution, and even after 24 h of immersion air is not completely replaced by the solution^22^. Thus, the replacement of air by Thoulet’s solution in NEC is expected to be a very slow process when the lesion is in this original physical presentation (within a whole crown as here). Such a relatively long duration process combined with the occurrence of surface layer in NEC with ICDAS score 1–2 renders only NEC with ICDAS score 3 suitable for profilometric analysis of surface changes in response to infiltration of dentinal fluid. The changes in surface height detected in our NEC lesions with ICDAS score 3 can be explained as a result of the penetration of dentinal fluid into the carious enamel pores, and then emerging on the NEC surface. These results raised the question on whether the dentinal fluid could interact with the biofilm fluid and affect the composition of a cariogenic biofilm formed *in vitro* on the NEC surface.

In our *in vitro* biofilm experiment, modified dentinal fluid was in contact with dentine for days, making it possible for the dentinal fluid to infiltrate the carious enamel pores and eventually reaches the outer enamel surface, where it interacted with the biofilm fluid. Such interaction resulted in the lowest amounts of EPS when 2% chlorexidine, known antibacterial agent on *S. mutans* biofilm^23^, was used to modify the dentinal fluid. The lower amounts of EPS in ECNaCl group compared to the groups with normal enamel can be explained by the persistence of residual quantities of chlorexidine in the NEC pores after the rapid wash of teeth with distilled water at the end of the first 5 days period (when chlorexidine was used). Considering the slow process of penetration of liquids with high penetration coefficient (Thoulet’s solution 1.47 has a penetration coefficient of 2297 cm/s^22^; while water has a penetration coefficient of 4039 cm/s) into dried NEC^22^, the rapid wash with water for 5 min is not expected to have replaced all the 2% chlorexidine solution in NEC pores.

The implications of our findings on the pathogenesis of PNEC rely on the likely interaction of the dentinal fluid with the pores of PNEC (Fig. 5) and with the overlying biofilm fluid. Interaction with carious enamel fluid might affect de- and remineralization and resin infiltration. Interaction with biofilm fluid might affect the virulence of the cariogenic biofilm, and, hence, affect de- and remineralization. Thus, our results also have implications on noninvasive treatment of PNEC.

This is particular important if one considers a wide range of dental restorative procedures that use chlorexidine/antibiotics-containing dental materials placed in the vicinity of dentine underlying PNEC. Chlorexidine and other antibiotics are found in the composition of intracanal irrigants and temporary endodontic medicaments^24^ and endodontic sealers^25^ used in root canal treatment, when dental pulp is removed (as it was the case in this study). Some conditions (eg: irreversible pulpitis, pulp necrosis, and apical periodontitis) require endodontic treatment with thorough cleaning up of the pulp and root canal obturation. While such a (necessary) treatment is quite invasive for the tooth, the endodontic/restorative materials used in endodontically-treated teeth might have a noninvasive effect on PNEC located in the remaining parts of the tooth crown. Components (eg: antibiotic and fluoride ions) released by such materials might diffuse through demineralized dentine and reach natural enamel caries and the overlying biofilm. In this context, it should be noted that chlorhexidine and fluoride are found in cavity liners (dental materials placed beneath the main restorative material)^26^ used in restorations of endodontically treated (pulpless) teeth and teeth with vital pulp and deep occlusal cavity.

As the main limitations of our study, the *in vitro* conditions used here lack the influence of dental fluids (i.e., saliva and periodontal fluid) that could exert an effect on the biofilm fluid and in the water content of dentinal tubules. The facilitated pathway for materials from the pulp chamber to the PNEC surface described here could be further explored *in vitro*, *in vivo*, or clinically. For the future, efforts on the study of suitable materials and or restorative protocols to optimize noninvasive effect of endodontic treatment on natural enamel caries should be pursued. Ongoing efforts to explore fluoride-releasing materials used as endodontic sealers are being conducted by our group.

In conclusion, this study evidenced that dentine subjacent to PNEC is mostly demineralized, providing facilitated pathway for dentinal fluid penetrate into PNEC and alter the composition of the biofilm formed on the PNEC surface.

## Electronic supplementary material

Insight of Captagon Abuse by Chemogenomics Knowledgebase-guided Systems Pharmacology Target Mapping Analyses

## Data Availability

Most of the data generated or analyzed during this study are included in this published article (and its Supplementary Information files). Data not found in this published data and its Supplemental files are available from the corresponding author on reasonable request.
