# Supplementary material for: Natural enamel caries, dentine reactions, dentinal fluid and biofilm
Source: Sci Rep. 2019 Feb 26;9:2841. doi: 10.1038/s41598-019-38684-7 (PMC6391475; doi:10.1038/s41598-019-38684-7)
Supplement: Supplementary file 1 — Insight of Captagon Abuse by Chemogenomics Knowledgebase-guided Systems Pharmacology Target Mapping Analyses [file 41598_2019_38684_MOESM1_ESM.docx]

**Natural enamel caries, dentine reactions, dentinal fluid and biofilm**

Laryssa de Barros Pinto^1^,

Maria Luiza Alves Lira^2^,

Yuri Wanderley Cavalcanti^3^

Eugênia Livia de Andrade Dantas^4^

Maria Lúcia Oliveira Vieira^5^

Gabriel Garcia de Carvalho^6^,

Frederico Barbosa de Sousa^7*^

1, 2, 4,5 Master Program in Dentistry, Health Sciences Center, Federal University of Paraiba, Joao Pessoa, Cidade Universitaria, 58051-900, Paraíba, Brazil.

^3^Department of Clinical and Social Dentistry, Health Sciences Center, Federal University of Paraiba, Joao Pessoa, Cidade Universitaria, 58051-900, Paraíba, Brazil.

^6, 7*^Department of Morphology, Health Sciences Center, Federal University of Paraiba, Cidade Universitária, 58051-900, João Pessoa, Paraiba, Brazil. Email: fredericosousa@hotmail.com

Key words: dental caries, dental enamel, dentine, dentinal fluid, histopathology, biofilm.

APPENDIX

Material and Methods

*Surface Changes of Carious Enamel in Response to Dentinal Fluid*

Surface of PNEC were analyzed in a 3D optical profilometer (CCI MP Profiler, Taylor Hobson, UK), with a 10X objective, under 50% of air relative humidity and 22 C. A filter of 0.08 μm was applied after scanning for processing of data from each surface, resulting in a map of surface height. A horizontal line was traced in the center of the image and mean surface height was measured. This procedure was repeated for every image in a time-series.

We tested the method error in detecting changes in mean surface height. The error was measured using measurements not expected to result in actual changes in mean surface height. For this, 48 proximal surfaces were analyzed for up to 30 min. Six of these (surfaces with ICDAS score 0) were analyzed before and after infiltration of Thoulet’s solution in the pulp chamber. The other 42 surfaces (6 with ICDAS score 0; 14 wit ICDAS score 1, and 22 with ICDAS score 2) were analyzed for 30 min, but no solution was applied in the pulp chamber. For each surface, 6 data points (baseline, 5 min, 10 min, 15 min, 20 min, and 30 min) were obtained, and the outcome was variation mean surface height in relation to the baseline value (ΔMSH). Error was calculated from root mean square of all ΔMSH values. The calculated error was multiplied to 3 yielding a value of 1.18 μm. This represents a statistical Z score of 3, with a 2-tailed probability of 0.27% for including an error in ΔMSH when the cutoff value was used.

*3D microcomputed tomography (microCT) analysis*

MicroCT analysis was performed in before and after the in vitro biofilm formation experiment. Teeth were scanned in a microCT (Skyscan 1172 model, Bruker, USA) using resolution of 13.8 μm (large size of pixel matrix), Al-Cu filter, rotation step of 0.4°, frame average of 4, random movement of 10, 100 kV, and 360° rotation. Reconstruction of 3D files was performed using smoothing correction of 4, beam hardening correction of 30%, and ring artifact correction of 4. After the biofilm experiment, the space between the plastic tube and the external root surface was filled with contrast solution in order to exclude samples with any leakage of the contrast solution to the tooth crown surface.

*Effect of Dentinal Fluid on the Biofilm Matrix Formed on the PNEC Surface*

Teeth with carious ICDAS 2 lesions were selected for this experiment. The two apical thirds of the root were removed from teeth in order to give full access to pulp chamber. Plastic micro-tubes had their bottom third cutted off. Teeth were then positioned upside down, in a way the cervical part of root were fixed in the bottom of plastic tube. Tooth/plastic interface was completed sealed. An acid resistant varnish was applied at the entire crown, except the region of ICDAS 2 lesions.

During the preparation of teeth for the in vitro biofilm formation, the tooth root surface was isolated form the tooth crown surface by a plastic micro-tube (with bottom third cutted off). One of the micro-tube ends was fixed on the cervix of the tooth using both a light cured composite resin and glue (superbond, 3M) in order to completely seal the tooth/plastic interface (Appendix Figure 1). With the tooth positioned upside (occlusal surface) down, the reservoir created between the root surface and the internal plastic micro-tube surface was filled with water and leakage was tested with absorbing paper applied on the crown side. Only samples with no leakage were included.


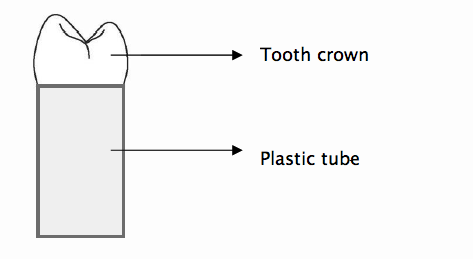


Appendix Figure 1. Isolation of tooth root surface from the tooth crown surface before the in vitro biofilm experiment.

Then, an acid resistant nail varnish (Maybelline, Paris) was applied on the entire tooth surface, except on a selected area on the proximal surface (Appendix Figure 2). This selected area was either the PNEC or normal enamel. Then, each set was submitted to sterilization with ethylene oxide.


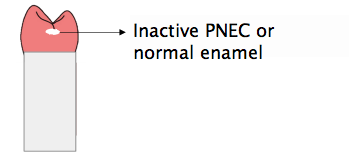


Appendix Figure 2. Coverage of tooth crown surface with nail varnish, leaning the selected area of enamel surface (either PNDC or normal enamel) available for in vitro of *S. mutans* biofilm iformation .

During in vitro biofilm formation, the root end was fixed in the back surface of a 15 mL centrifuge tube cap using an orthodontic wire and light cured composite resin. With the tooth upside (occlusal surface) down (bottom of the centrifuge tube), two reservoirs were created: the pulp chamber (where 2% chlorexidine or 0.9% NaCl solutions was inserted; reservoir connected with outer root surface), and the bottom part of the centrifuge tube (where 2 mL of culture medium of tryptone and yeast extract broth added with 1% sucrose was inserted).

Sterilized teeth fixed on plastic micro-tubes were positioned inside 15 mL tubes for biofilm growth. *S. mutans* (UA 159) strain was used to develop biofilms on the enamel surface. Initially, a standard suspension of *S. mutans* was prepared to obtain at 1 x 10^8^ colony forming units per milliliter. This was equivalent to an absorbance of 0.1 at 600 nm. Biofilm was formed in the presence of 2 mL of tryptone and yeast extract broth^1^ supplemented with 1% sucrose, for 96 h. The culture medium was replaced every 24 h.

Immediately before biofilm formation, 1 mL of 0.09% NaCl solution or 2% chlorhexidine solution was inserted within the plastic tube fixed to teeth, in way that solutions perfused pulp chamber. It was expected saline would not interfere with biofilm formation, whilst chlorhexidine would inhibit the production of extracellular polysaccharides (EPS). The whole experiment was performed four times, alternating normal enamel and PNEC in the selected area for biofilm formation.


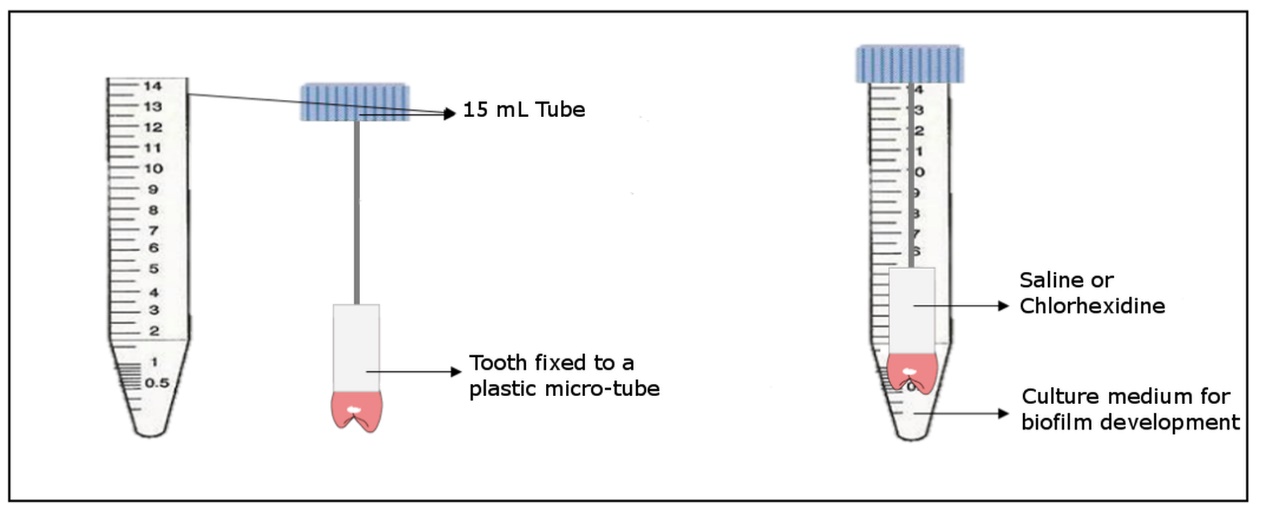


Appendix Figure 3. Illustration how teeth with exposed selected enamel area were fixed to a plastic tube and settled up for biofilm formation.

After 96 h of biofilm formation (5 days), the culture medium was removed. Biofilm attached to crown surface were removed by vortexing with 1 mL saline. Suspensions of biofilms were used to extract EPS^2-3^. Biofilm suspensions were centrifuged (500 g) for 5 min. Soluble EPS was precipitated with 99% ethanol from biofilm’s supernatant. Insoluble EPS was extracted from biofilm’s pellet using 1% NaOH solution, followed by precipitation with 99% ethanol. Soluble and insoluble EPS were re-suspended with 250 uL of 1% NaOH and dosage was made through the phenol-sulfuric method^4^. Briefly, 200 uL of suspended EPS was added to 200 uL of 5% phenol and 1 mL of sulfuric acid. Spectrophotometer readings at 490 nm were run and absorbances of samples were interpolated with a standard curve of known concentrations of glucose.

*Statistical analysis*

Sample size calculations

All procedures followed equations provided in the literature^5^. Sample size calculations were performed for three experiments, taking into account their respective null hypotheses. For the real-time tracking of the transport of dentinal fluid towards PNEC, we considered a one-sample test with difference between two proportions of lesions with facilitated transport towards PNEC: the estimated proportion of 20% (considered as small) and the experimental proportion measured. The proportion of 20% can be considered small since 20% of pooled differences between groups is considered small in statistics^5^. The null hypothesis was that facilitated transport from the pulp chamber to PNEC did not occur in more than 20% of the teeth. As there was no published data on the expected effect size, the value of medium effect size for the difference between proportions (Cohen H effect size of 0.5) was used, as recommended^5^, along with a 1-tailed 5% significance level, and 80% of power, resulting in a sample size of 49 lesions. Using an estimated sample loss of 15%, final calculated sample size was 56.

For the experiment of differences between correlations coefficients (correlation between enamel and dentine reactions using SM for dentine reactions compared with the correlation between enamel and dentine reactions using MRC for dentine reactions), it was also considered a medium effect size (Cohen effect size q of 0.42, for differences between correlations coefficients), because at the time of the planning of this study there was no similar studies published in the literature. The published correlation of 0.87 between enamel and dentine reactions using SM for dentine reactions^6^ was used for the one correlation, and it should differ from the other correlation with a Cohen q effect size of 0.42. Considering this, a 5% 1-tailed significance level, and power of 80%, the calculated sample size was 70 lesions. Using an estimated sample loss of 30%, final calculated sample size was 91 lesions (one per tooth).

For the experiment of in vitro biofilm formation on the PNEC surface, it was used an effect size Hedge’s g of 2 (from a pilot study), a 5% 2-tailed significance level, and power of 80%, resulting in a calculated sample size of 6. Using an estimated loss of 60% (due to the selection criteria), final calculated sample size was 10 lesions.

Post hoc analysis

When evaluating assumptions for ANOVA, homogeneity of variances was not tested because ANOVA is only slightly affected by heterogeneous variances when groups have equal sample size as it was here^7^. Following recommendations for post hoc comparisons of pairs of groups when all comparisons are planned in advance^8^, it was applied the paired T test without correction for type I error. For normality test, data with skewness between -2 and +2, and kurtosis between 1 and 5 were considered as normally distributed^9^.

Repeated measures ANOVA (5% significance level) and post hoc paired T test (1-tailed 5% significance level) were also applied to test the hypothesis that the dentinal fluid affects the ratio of insoluble EPS to soluble EPS in the *S. mutans* biofilm formed on the PNEC surface.

**Results and Discussion**

*Surface Changes of Carious Enamel in Response to Dentinal Fluid*

Changes in mean surface height on the PNEC (ICDAS 3; Nyvad score of inactive) surface in response to a solution applied in the pulp chamber are shown in Appendix Figure 4. Variations up to 30 min after infiltration of the liquid in the pulp chamber are shown. Each sample presents pronounced (outside the error) changes in more than one moment in the time series. It could be detected that changes are located mainly in areas with more intense surface breakdown (Appendix Figure 5).

Appendix Figure 4. Plot of variations in mean surface height on the surface of four PNEC lesions (ICDAS score 3; Nyvad score of inactive) over time after infiltration of Thoulet`s solution in the pulp chamber.


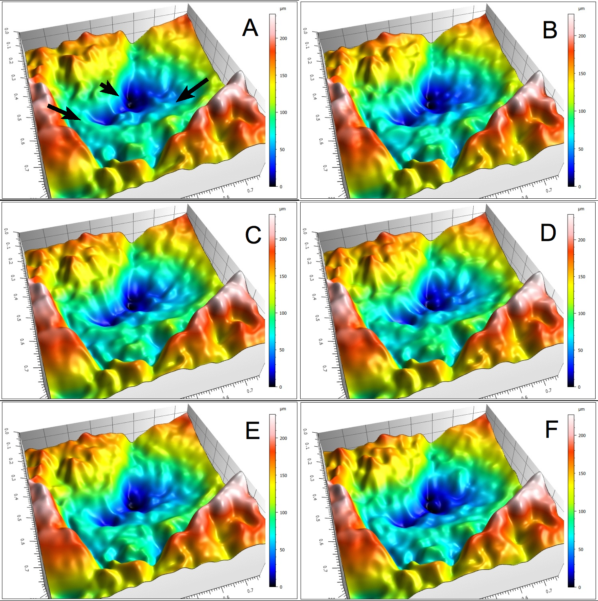


Appendix Figure 5. Changes in the surface height of PNEC (ICDAS score 3; inactive) after infiltration of Thoulet’s solution in the pulp chamber. A-D are images taken at baseline and after 5 min, 10 min, 15 min, 20 min, and 30 min of infiltration of the pulp chamber. Black arrows indicate areas of marked changes (see change in surface color).

*Correlation between enamel and dentine histological reactions to caries*

In this experiment, distribution of proximal surface according to ICDAS scores was as follow: 5 (5.5%) with ICDAS score 0, 4 (4.4%) with ICDAS score 1, 72 (79.1%) with ICDAS score 2, and 10 (11%) with ICDAS score 3.

Data on enamel and dentine histological scores (from both SM and MRC) are shown in Appendix Table 1. The correlations between enamel and dentine reactions to caries are shown separately, for both SM and MRC as techniques used for dentine reactions, in Appendix Figure 6.

Appendix Table 1. Histological scores for enamel and dentine reactions to caries. Dentine scores measured under SM are between parentheses (SM), and those measured under MRC are between brackets [MRC].

| Enamel  scores | Dentine scores | | | | |  |
| --- | --- | --- | --- | --- | --- | --- |
|  | D1 | D2 | D3 | D4 | D5 | Total |
| E1 | (05) [01] | (37) [23] | (00) [00] | (00) [00] | (00) [18] | 42 (46,15%) |
| E2 | (00) [00] | (08) [02] | (00) [01] | (00) [00] | (00) [05] | 8 (8,8%) |
| E3 | (00) [01] | (12) [10] | (03) [01] | (13) [05] | (01) [12] | 29 (31, 9%) |
| E4 | (00) [00] | (03) [00] | (00) [01] | (06) [02] | (01) [07] | 10 (11%) |
| E5 | (00) [00] | (01) [00] | (00) [00] | (01) [00] | (00) [02] | 2 (2,2%) |
| Total | (05) [02] | (61) [35] | (03) [03] | (20) [07] | (02) [44] | 91 (100%) |


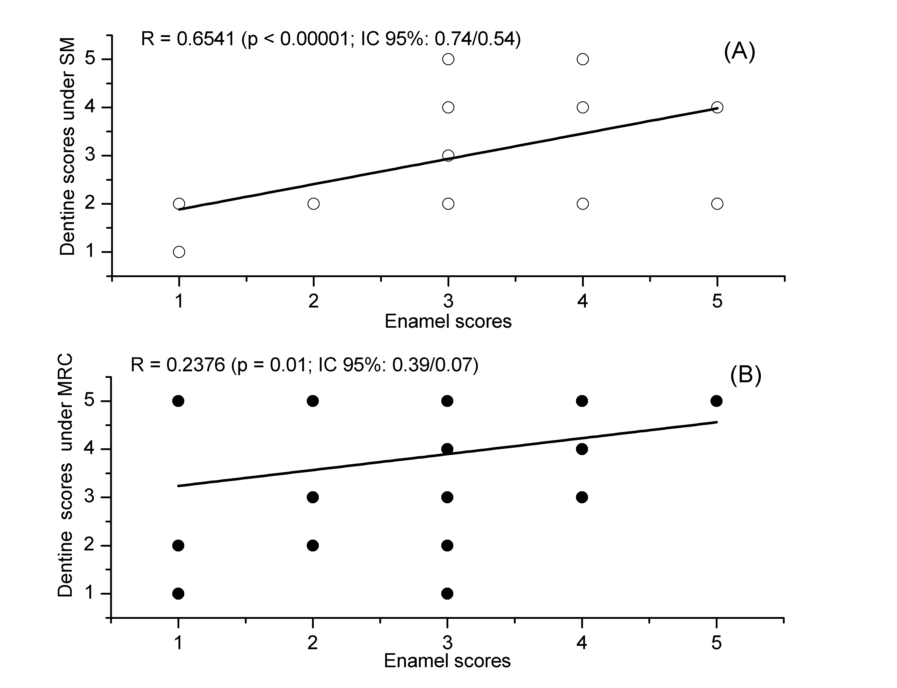


Appendix Figure 6. Plots of the correlations between enamel and dentine reactions to caries using SM (A) and MRC (B) for detecting dentine reactions.

*Effect of Dentinal Fluid on the Biofilm Matrix Formed on the PNEC Surface*

Typical aspect of *S. mutans* biofilm pellet after centrifugation is shown in Appendix Figure 7. The results of the statistical analysis (repeated measures one-way ANOVA) of the effect of the dentinal fluid on the ratio of soluble EPS to insoluble EPS of *S. mutans* biofilm indicate that the null hypothesis was rejected: p = 0.0062, effect size η^2^ = 27.19%, and power of 0.874. Descriptive statistics and pair-wise comparison between groups are shown in Appendix Table 2. Only the use of 2% chlorexidine resulted in conclusive differences in the ratio of insoluble EPS to soluble EPS. This ratio is more important in the cariogenic potential of biofilms than the amounts of soluble and insoluble EPS separately^10^. EPSi play a wider contribution than EPSs to the cariogenic potential of *S. mutans* biofilm^10^.


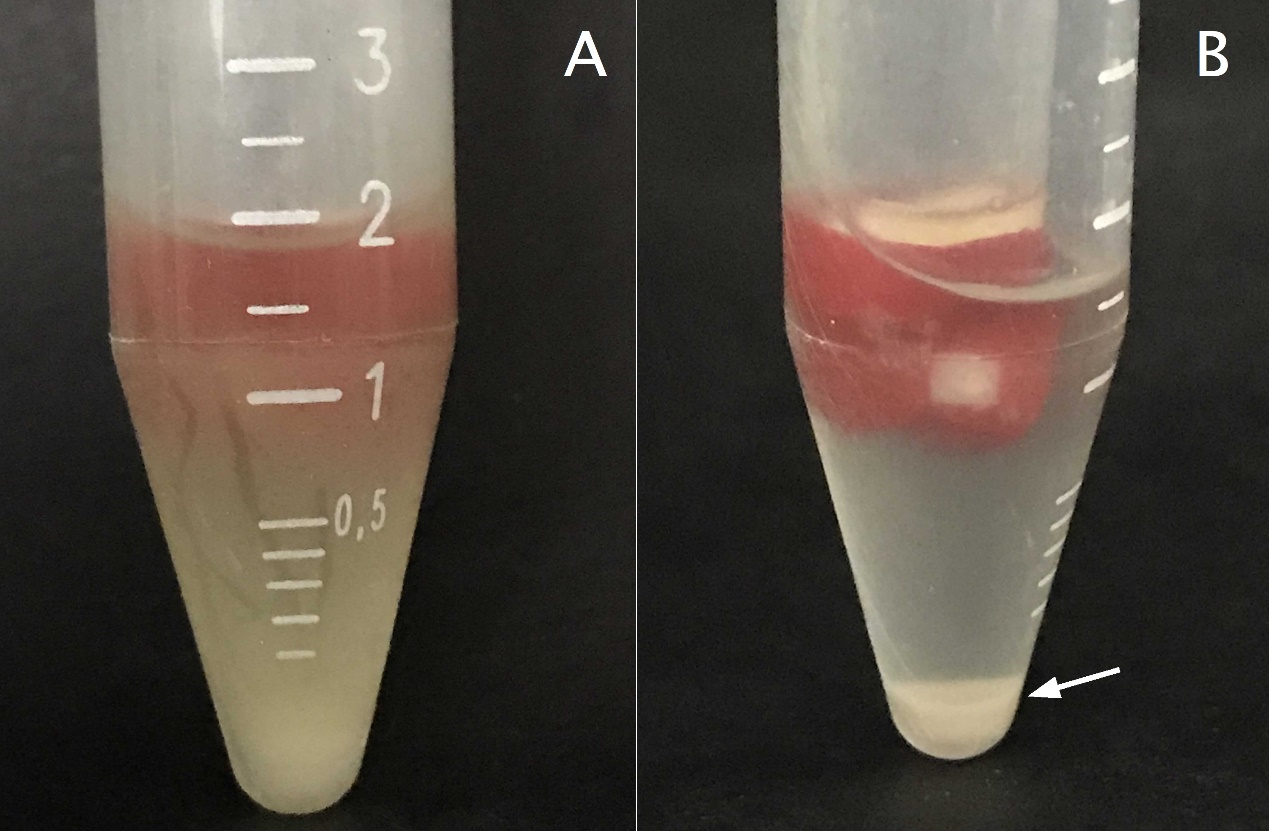


Appendix Figure 7. Samples after the 5 days period of biofilm formation (ECNaCl group). A, before removal of culture medium. B, after removal of culture medium, insertion of saline solution, and centrifugation, with biofilm pellet in the bottom part (arrow).

Appendix Table 2. Results of the effect of the dentinal fluid on the ratio of soluble EPS to insoluble EPS of *S. mutans* biofilm formed in vitro on enamel surface.

|  | Groups | | | |
| --- | --- | --- | --- | --- |
|  | ECChl | ECNaCl | NEChl | NENaCl |
| EPSi/EPSs ratio – Mean (SD) | 0.509 (0.384) | 1.203 (0.299) | 0.925 (0.536) | 0.892 (0.426) |
| *Post hoc paired T tests (5% 1-tailed)*  *Soluble EPS* |  |  |  |  |
| Groups compared | P value | Hedge’s g | 95% CI | Power |
| ECChl X ECNaCl | 0.0009 | 1.38 | 2.29/ 0.47 | 0.984 |
| ECChl X NEChl | 0.0273 | 0.70 | 1.54/ -0.14 | 0.641 |
| ECChl X NENaCl | 0.0265 | 0.70 | 1.55/ -0.14 | 0.644 |
| ECNaCl X NECChl | 0.1122 | 0.49 | 0.35/ -1.32 | 0.004 |
| ECNaCl X NENaCl | 0.0465 | 0.59 | 0.25/ -1.42 | 0.002 |
| NEChl X NENaCl | 0.4225 | 0.06 | 0.76/-0.88 | 0.036 |

**References**

1. Cavalcanti, Y.W., Bertolini, M.M., da Silva, W.J., Del-Bel-Cury, A.A., Tenuta, L.M. & Cury, J.A. A three-species biofilm model for the evaluation of enamel and dentin demineralization. *Biofouling* **30**(5), 579-88 (2014).

2. Aires, C.P., Del Bel Cury, A.A., Tenuta, L.M.A., Klein, M.I., Koo, H., Duarte, S. & Cury, J.A. Effect of starch and sucrose on dental biofilm formation and on root dentine demineralization. *Caries Res*. **42**, 380–386 (2008).

3. Martins, M.L., Leite, K.L.F., Pachecho-Filho, E.F., Pereira, A.F.M., Romanos, M.T.V., Maia, L.C., Fonseca-Gonçalves, A., Padilha, W.W. & Cavalcanti, Y.W. Efficacy of red propolis hydro-alcoholic extract in controlling Streptococcus mutans biofilm build-up and dental enamel demineralization. *Arch. Oral Biol*. **93**(2): 56-65 (2018).

4. Dubois, M., Gillis, K.A., Hamilton, J.K., Rebers, P.A. & Smith, F. Colorimetric method for determination of sugars and related substances. *Analyt. Chem*. **28**, 350–356 (1956).

5. Cohen, J. Statistical power analysis for the behavioral sciences. (Lawrence Erlbaum Associates, 1988).

6. Bjorndal, L. & Thylstrup, A. A structural analysis of approximal enamel caries lesions and subjacent dentin reactions. *Eur. J. Oral Sci.* **103**(1), 25-31 (1995).

7. Kutner, M.H., Christopher, J., Nachtsheim, J.N. & William L. Applied Linear Statistical Models. (Mcgraw Hill, 2004).

8. Armstrong, R.A. When to use the Bonferroni correction. *Ophthalmic Physiol. Opt*. **34**(5), 502-508 (2014).

9. Field, A. Discovering Statistics Using SPSS. (SAGE, 2009).

10. Cury, J. A., Rebelo, M. A. B., Cury, A. D. B., Derbyshire, M. T. V. C., & Tabchoury, C. P. M. Biochemical composition and cariogenicity of dental plaque formed in the presence of sucrose or glucose and fructose. *Caries Res*. **34**(6), 491-497 (2000).
